# *In Vivo*-to-*In Silico* Iterations to Investigate Aeroallergen-Host Interactions

**DOI:** 10.1371/journal.pone.0002426

**Published:** 2008-06-11

**Authors:** Alba Llop-Guevara, Marc Colangelo, Derek K. Chu, Cheryl Lynn Moore, Nicole A. Stieber, Tina D. Walker, Susanna Goncharova, Anthony J. Coyle, Lennart K. A. Lundblad, Paul M. O'Byrne, Miroslav Lovric, Manel Jordana

**Affiliations:** 1 Department of Pathology and Molecular Medicine, Division of Respiratory Diseases and Allergy, Centre for Gene Therapeutics, McMaster University, Hamilton, Ontario, Canada; 2 Department of Inflammation and Autoimmunity, MedImmune Inc., Gaithersburg, Maryland, United States of America; 3 Department of Medicine, McMaster University, Hamilton, Ontario, Canada; 4 Department of Mathematics and Statistics, McMaster University, Hamilton, Ontario, Canada; 5 Vermont Lung Center, University of Vermont, Burlington, Vermont, United States of America; Centre de Recherche Public-Santé, Luxembourg

## Abstract

**Background:**

Allergic asthma is a complex process arising out of the interaction between the immune system and aeroallergens. Yet, the relationship between aeroallergen exposure, allergic sensitization and disease remains unclear. This knowledge is essential to gain further insight into the origin and evolution of allergic diseases. The objective of this research is to develop a computational view of the interaction between aeroallergens and the host by investigating the impact of *dose* and *length* of aeroallergen exposure on allergic sensitization and allergic disease outcomes, mainly airway inflammation and to a lesser extent lung dysfunction and airway remodeling.

**Methods and Principal Findings:**

BALB/C mice were exposed intranasally to a range of concentrations of the most pervasive aeroallergen worldwide, house dust mite (HDM), for up to a quarter of their lifespan (20 weeks). Actual biological data delineating the kinetics, nature and extent of responses for local (airway inflammation) and systemic (HDM-specific immunoglobulins) events were obtained. Mathematical equations for each outcome were developed, evaluated, refined through several iterations involving *in vivo* experimentation, and validated. The models accurately predicted the original biological data and simulated an extensive array of previously unknown responses, eliciting two- and three-dimensional models. Our data demonstrate the non-linearity of the relationship between aeroallergen exposure and either allergic sensitization or airway inflammation, identify thresholds, behaviours and maximal responsiveness for each outcome, and examine inter-variable relationships.

**Conclusions:**

This research provides a novel way to visualize allergic responses *in vivo* and establishes a basic experimental platform upon which additional variables and perturbations can be incorporated into the system.

## Introduction

Allergic asthma emerges from the interaction between two complex dynamic systems, the immune system and the environment, where aeroallergens exist. These systems are intricate, comprise multiple parts which are subject to many interactions and feedback loops and, consequently, contain a broad array of outputs. The interaction between these already complex systems generates an even higher degree of complexity. Thus, deciphering the conditions under which allergic disease evolves would benefit from the elaboration of models that can explain and/or predict the potential outputs of that interaction.

Advances in the understanding of disease processes have come in great measure through experimentation using *in vitro* and, notably, *in vivo* human and animal models. A detailed appreciation of the immunopathology of asthma, along with the explosion in molecular immunology has prescribed the modeling strategies to recapitulate the asthmatic phenotype, particularly in mice. It should be noted that conventional biomedical modeling greatly differs from modeling in other scientific domains, such as ecology or economics in that biomedical models are conceived with a pre-established goal in mind: to establish a known phenotype. While such an approach has produced conspicuous benefits, it has inherently prevented an unbiased, global understanding of the consequences of the interaction between allergens and the immune system.

Although it is generally thought that there is a reasonable correlation between early allergen exposure and sensitization [1], [2], [3], [4] or sensitization and disease [1], [5], [6], the connection that may exist between exposure and disease is less clear [1]. The intrinsic constraints of these clinical and epidemiological studies preclude achieving both a longitudinal and quantitative understanding of these relationships. Yet, it seems intuitive that such knowledge is essential to gain further insight into the origin, evolution and nature of allergic disease.

The strategy that we followed to investigate the relationship between aeroallergen exposure, allergic sensitization and allergic disease embraces a computational conception of immune responsiveness [7]. In this conception, the view is *synthetic* rather than *analytical* and, therefore, the focus is on system behaviors rather than specific components, i.e. the complex molecular networks underlying the outcomes that we measured. We surmise that this strategy is justified *ad interim* given the current state of knowledge in systems biology *in vivo*. We present data delineating the kinetics and dose-responses for local (total inflammation and eosinophilia) and systemic (HDM-specific immunoglobulins (Ig) G_1_ and E) events elicited in mice by extended exposure to house dust mite (HDM). We developed and refined algorithms defining the behavior of each outcome that were subsequently used to conduct *in silico* simulations to guide new biological experiments and visualize an extensive array of unknown responses. We propose that the iterative approach applied to construct the model exhibits considerable fidelity to the biological structure of the process.

## Methods

### Animals

Female BALB/C mice (6 to 8 weeks old) were purchased from Charles River Laboratories. The mice were housed in a specific pathogen-free environment under 12 h light-dark cycle. All experiments described in this report were approved by the Animal Research Ethics Board of McMaster University.

### Protocol of respiratory mucosal sensitization

House dust mite extract (Greer Laboratories) was resuspended in saline (0.9% NaCl Irrigation Solution, Baxter) and serial dilutions were done to obtain the desired concentrations. This suspension was delivered to isoflurane-anaesthetized mice intranasally in a 10 µl volume. Mice were exposed daily to HDM for either 10 consecutive days (short-term protocol) or 5 consecutive days a week followed by 2 days of rest for a total of 1, 2, 3, 5, 7, 10, 14 and 20 weeks (long-term protocol).

### Sample collection

At various time-points, always 72 hours after the last HDM exposure, mice were sacrificed. Blood was collected by retro-orbital bleeding. Blood smears where prepared and serum was obtained by centrifugation of whole blood. Bronchoalveolar lavage (BAL) was performed as previously described [8], [9]. Briefly, the lungs were dissected, the trachea was cannulated with a polyethylene tube (BD Biosciences) and two lavages were done with PBS (0.25 ml followed by 0.2 ml). Total cell counts were then determined using a hemocytometer and smears were prepared by cytocentrifugation. Protocol Hema 3 stain set (Fisher Scientific) was used to stain blood and BAL smears and differential cell counts (≥500 leukocytes) were determined according to a previously established protocol [9]. The right lobe of the lung was inflated and fixed in 10% formalin for histological analysis.

### HDM-specific Ig measurements

Levels of HDM-specific IgE and IgG_1_ in serum were measured using ELISA techniques as previously described in detail [10]. Optical density (OD) was read at 405 nm. HDM-specific IgE titres (in OD units) were calculated by subtracting from each sample OD the average OD value of 20 zero standard replicates plus two standard deviations. HDM-specific IgG1 titers (in relative units) were calculated using the formula 1/(*x*/ODx*0.05), where *x* equals the dilution factor closest to but greater than double the average OD value of 20 zero standard replicates, and OD*x* is the OD reading of *x*.

### Determination of airway responsiveness

Mice were anesthetized with nebulized isoflurane (3% with 1 L/min of O_2_), paralyzed with pancuronium bromide (1 µg i.p.), tracheostomized with a blunted 18-gauge needle, and mechanically ventilated with a small animal computer-controlled piston ventilator (flexiVent, SCIREQ Inc.) [11]. Mice received 200 breaths per minute and a tidal volume of 0.25 ml; the respiratory rate was slowed during nebulization (10 seconds) to provide 5 large breaths of aerosol at a tidal volume of 0.8 ml. The response to nebulized saline and increasing doses (3.125, 12.5 and 50 mg/ml) of methacholine (MCh, Sigma-Aldrich) was measured. A positive-end-expiratory pressure of 3 cm of H_2_O was applied by submerging the expiratory line in water. Respiratory impedance was determined from 3 second broadband volume perturbations ranging from 1 to 20.5 Hz every 10 seconds during approximately 2 minutes following each dose of MCh. The data was fitted with the constant phase model and model parameters (airway resistance (Rn), tissue dampening (G), tissue elastance (H) and hysteresivity, a measure of lung heterogeneity (η = G/H)) were calculated [12]. Model fits that resulted in a coefficient of determination less than 0.8 were excluded.

### Histology and morphometric analysis

Lung tissue was embedded in paraffin and cut at a thickness of 3 µm. Sections were stained with hematoxylin and eosin for evaluation of the severity and the nature of leukocyte infiltration in the lungs by light microscopy. Additional sections were stained with Picro Sirius red to demonstrate the presence of collagen in the extracellular matrix. Images of main airways were captured with OpenLab (Improvision) via a Leica camera and microscope attached to a computer. Analysis was performed on a custom computerized image analysis system (Northern Eclipse software, Empix Imaging) as previously described [13]. Briefly, morphometric quantification involved calculation of the percentage of tissue area that was positively stained within a 40 µm-thick area from the basement membrane extending into the airway lumen.

### Mathematical and computational modeling

All the equations for the mathematical models and analyses were generated using curve-fitting techniques within FindGraph software (UNIPHIZ Lab) for each outcome. (All equations and additional detail on the validation analysis of the model is provided in an online data supplement)

### Data analysis

Data are expressed as means±standard error of the mean (s.e.m.). Statistical analysis was performed with GraphPad Prism (GraphPad Software). Results were interpreted by analysis of variance (one-way ANOVA) followed by the Dunnett *post hoc* test to compare HDM exposed groups versus the saline control group. Differences were considered statistically significant when p values where less than 0.05.

## Results

### Dose-response to short-term HDM exposure

**Figure 1:**
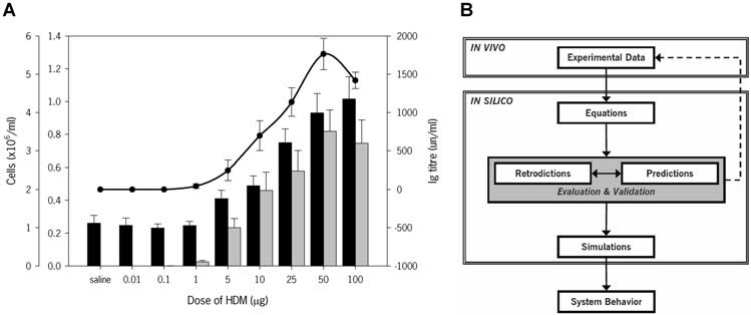
Airway inflammation and systemic immunity in BALB/C mice exposed to HDM for 10 consecutive days. (A) Dose-response: total inflammation (black bars), eosinophilia (grey bars) and serum HDM-specific IgG_1_ (solid circles and solid line). Results for cells (n = 5–19 mice/group) and IgG_1_ (n = 2–6 mice/group) are expressed as means±s.e.m. (B) Schematic of the steps followed to develop the mathematical models.

We have previously shown that mice exposed intranasally to HDM at a concentration of 25 µg/day for 10 consecutive days develop acute airway inflammation [14], and that exposure to 25 µg/day for up to 7 weeks establishes chronic airway inflammation associated with remodeling [15]. These static conditions, fixed times and concentrations, were selected to achieve desired specific outcomes, thus neglecting the dynamic nature of a living system. Hence, we initially carried out a dose-response experiment using a 10,000-fold range, from 0.01 to 100 µg/day, for 10 days. As shown in Figure 1A, the response in terms of total cell numbers (TCN), eosinophils (EOS) and HDM-specific serum IgG_1_ followed a logistic pattern with an incipient response observed with 1 µg and a plateau after 25 µg. Based upon these findings, we chose doses of 1 (incipient), 7.5 (moderate) and 25 µg/day (submaximal) for subsequent experiments utilizing longer exposure periods.

### Modeling the inflammatory response to long-term HDM exposure

**Figure 2:**
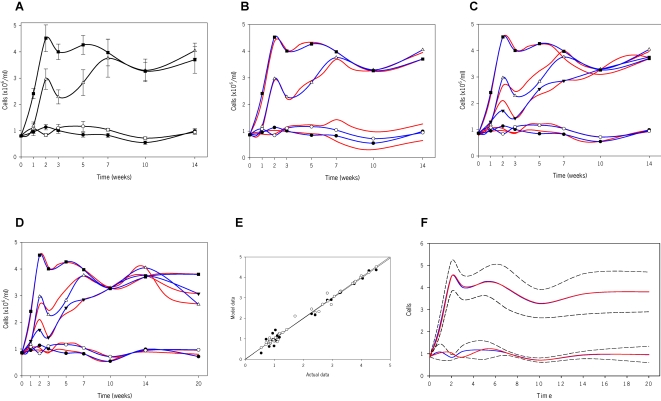
Airway inflammation in BALB/C mice exposed to HDM and subsequent mathematical modeling. (A) Inflammatory response in the BAL. Mice were exposed intranasally to either saline (solid circles) or HDM, 1 µg (open circles), 7.5 µg (open triangles up) or 25 µg (solid squares) for up to 14 weeks (5 days of exposure and 2 days of rest per week). Cell numbers are expressed as mean±s.e.m (n = 6–12 mice/group). (B) Mathematical modeling of the inflammatory response. A mathematical equation was developed from the experimental results (blue lines) based on dose and length of exposure to HDM. Simulations (red lines) for each of the doses studied experimentally were generated. (C) Results of the first iteration. Predictions using the first mathematical model were generated for 5 µg of HDM (see Figure S1) and were subsequently evaluated experimentally. Then, the new 5 µg experimental data (solid triangles down) was used to readjust the previous equation and refine the model. Actual data (blue lines) and virtual simulations (red lines) are shown for saline, 1, 5, 7.5 and 25 µg of HDM up to 14 weeks. (D) Results of the second iteration. Predictions using the second mathematical model were generated for all doses studied at 20 weeks of exposure (not shown) and were subsequently evaluated experimentally. Then, the new 20 week data was used to readjust the previous equation and further refine the model. Actual data (blue lines) and virtual simulations (red lines) are shown for saline, 1, 5, 7.5 and 25 µg of HDM up to 20 weeks. (E) Regression analysis to evaluate the accuracy of the mathematical models. Deterministic validation metrics were performed to mathematically measure the agreement between computational predictions and experimental results. For the line y = x, where y is model data and x is actual data, the coefficient of determination R^2^ in the first model is 0.987 (solid circles) and 0.990 in the last one (open circles). (F) Confidence intervals (CI) to evaluate the accuracy of the mathematical models. Non-deterministic validation metrics were also used to account for experimental and computational uncertainties and errors. The 95% CI for each point depicts uncertainty due to experimental variability. Model data for 25 µg (top) and 1 µg (bottom) accurately predict actual data, and fall within the 95% CI band.

We investigated the impact of exposing mice to those three concentrations of HDM for up to 14 weeks. As shown in Figure 2A, repeated allergen exposure initially elicited airway inflammation in a near-exponential manner that was both dose- and time-dependent. At 2 weeks, 7.5 and 25 µg led to a distinct peak in inflammation; from then on, 25 µg maintained a stable level of maximal inflammation. A similar plateau was also achieved with 7.5 µg only after 7 weeks. Interestingly, exposure to 1 µg of HDM even for such a protracted period of time did not elicit significant airway inflammation suggesting that a threshold of responsiveness for this outcome must be above this concentration.

Once experimental data were collected and analyzed, a *bottom-up* model was constructed using mathematical and computational methods to accurately portray the data and the ensuing dynamics. Although identical methodology was used for all outcomes (TCN, EOS and immunoglobulins), detailed steps (Figure 1B) are presented for only TCN for brevity.

The initial equation was encoded to be used iteratively to simulate and predict output responses given inputs of dose and time. Figure 2B shows a *retrodiction* of the model in which simulations were compared to actual data and proved to fit fairly well. Given that lower doses (saline and 1 µg) exhibited a seemingly different behavior compared to higher doses (7.5 and 25 µg), an intermediate dose of 5 µg was selected to further assess the model. In this evaluation, a *prediction*, or interpolation, of a 5 µg dose was performed and compared to actual data (Figure S1A). Figure 2C depicts a refined model that incorporates these new experimental data.

An additional evaluation of the model was also carried out by extrapolating data up to 20 weeks (Figure S1B). Following comparison to actual data, the model was further refined to include all experimental data (Figure 2D), resulting in a final equation generated from 5 doses and 9 time-points. Equation 1 describes total inflammation (*y*) as a function of time (*t*) and dose (*x*), while each of the respective co-efficients (*x_a1_*, *x_b1_*…) represent dose-dependent quantities (see Methods S1, equations 1.1 to 1.21):
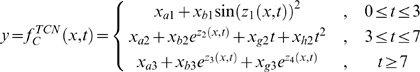
(1)


While the model visually fit the experimental data, accuracy was verified and quantified mathematically. Using linear regression, the initial equation, the revised equation (including the 5 µg data), and the final equation (including both the 5 µg and 20 week data) yielded R^2^ values of 0.987, 0.987 (not shown), and 0.990, respectively (Figure 2E). Furthermore, 95% confidence intervals (CI) and global validation metrics were calculated (see online data supplement and Figure 2F). The latter accounts for experimental uncertainty, error and chance [16], [17], [18], and confirmed that not only the model accurately predicts actual responses but also that an additional dose in the model did not enhance the accuracy of the system, while the integration of a further time-point had only a minimal effect.

This *complex* model accurately mimics varying system dynamics. However, in order to facilitate the visualization of responses, we developed a *simple* model (see online data supplement), represented by Equation 2, which captures the general features of the system:
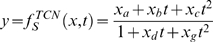
(2)


**Figure 3:**
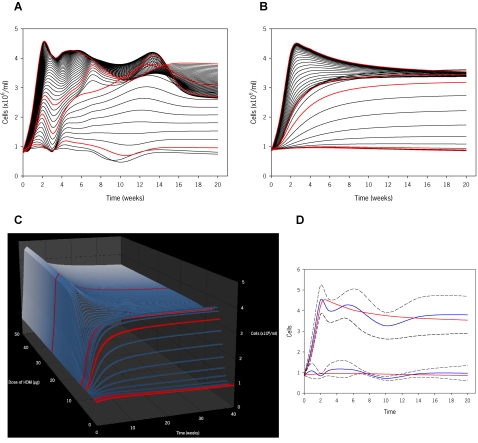
2D and 3D models for total cells generated from mathematical equations. In panels A, B and C, the simulations for the doses of HDM used experimentally are highlighted in red. (A) Simulations based on dose of HDM (range from 0 to 25 µg at 0.5 µg intervals) and length of exposure (0 to 20 weeks) using the final mathematical model (Figure 2D). (B) Simplified mathematical model for the total cell number. (C) 3D simulations generated from the simplified mathematical model including predictions up to 50 µg and 40 weeks of HDM exposure. (D) Confidence intervals to evaluate the accuracy of the simple mathematical model. Visual inspection shows that the simple model falls within the 95% CI, while quantification of the simple TCN model accuracy was calculated to be 89.03±22.42% with 95% confidence. Thus, the simple TCN model has similar fidelity to the complex model.

To visualize the dynamics of the system, simulations were performed with both *complex* and *simple* models to emulate the response between 0 and 25 µg, in sequential 0.5 µg increments over a 20 week period (Figure 3A–B). Further extrapolations were performed doubling both the highest dose and latest time-point used to construct the model. Figure 3C shows simulations in three-dimensions up to 40 weeks (approximately 50% of the lifespan of a mouse) and 50 µg to further enhance the visualization of the dose-time-response relationship. The *complex* model predicts actual data with slightly greater accuracy than the *simple* model, as indicated by an approximate 0.05 increase in the R^2^ value and an 8.5% increase in the 95% CI (data not shown and Figure 3D). Such simulations epitomize visual and numerical information that can only be derived mathematically and computationally.

**Figure 4:**
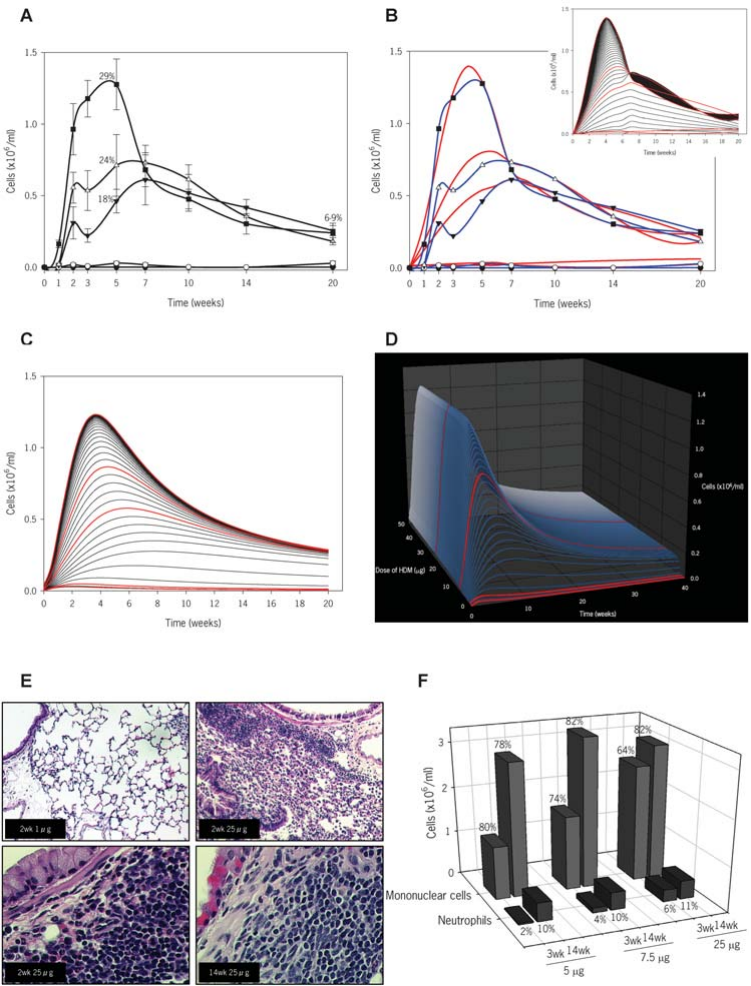
Nature of the inflammatory response in mice exposed to HDM and mathematical modeling of eosinophils. In panels B *insert*, C and D, the simulations for the doses of HDM used experimentally are highlighted in red. (A) Eosinophilic response in the BAL fluid. Mice were exposed to either saline (solid circles) or HDM, 1 µg (open circles), 5 µg (solid triangles down), 7.5 µg (open triangles up) or 25 µg (solid squares) for up to 20 weeks. Eosinophil numbers are expressed as mean±s.e.m (n = 6–12 mice/group); percentage of eosinophils at 5 and 20 weeks are inserted in the graph. (B) Final mathematical model for eosinophils. The equation to obtain these predictions (red lines) was developed from the experimental results (blue lines) based on dose and length of exposure to HDM. The *insert* shows simulations based on dose of HDM (range from 0 to 25 µg at 0.5 µg intervals) and length of exposure (0 to 20 weeks) using the final mathematical model. (C) Simplified mathematical model for eosinophils. (D) 3D simulations generated from the simplified mathematical model including predictions up to 50 µg and 40 weeks of HDM exposure. (E) Light photomicrograph of lung sections stained with hematoxylin and eosin. Top left: after 2 weeks of exposure to 1 µg of HDM (×10 magnification); top right: after 2 weeks of exposure to 25 µg (×10); bottom left: after 2 weeks of exposure to 25 µg of HDM (×40); bottom right: after 14 weeks of exposure to 25 µg of HDM (×40). (F) Cellular profile in the BAL fluid. Absolute numbers and percentage of mononuclear cells (light grey bars) and neutrophils (dark grey bars) after continued exposure to 5, 7.5 and 25 µg of HDM for either 3 or 14 weeks. Bars represent mean of cells (n = 6–12 mice/group).

Analysis of global inflammation was followed by an evaluation of airway eosinophilia, a typical hallmark of allergic inflammation. As illustrated in Figure 4A, eosinophils initially increase in a dose- and time-dependent fashion but later dramatically decrease to a level of 6–9% of total cells at 20 weeks of exposure. These findings are supported by a histopathological assessment (Figure 4E). As shown in the top panels, inflammation at 2 weeks is minimal in mice exposed to 1 µg of HDM and severe in those exposed to 25 µg. A graded level of inflammation was evident after exposure to 5, 7.5 and 25 µg (data not shown). Figure 4E (bottom panels) depicts a comparison of acute versus chronic exposure revealing stable inflammation over time but a relative decrease in tissue eosinophilia at later time-points. To note, we also observed a similar decrease in eosinophils in peripheral blood (data not shown). At variance with these findings, neutrophils and, particularly, mononuclear cells increased throughout the entire duration of allergen exposure, numerically compensating for the decrease of eosinophils and, hence, maintaining the overall degree of inflammation (Figure 4F). Again, exposure to 1 µg of HDM for up to 14 weeks did not elicit any significant changes in eosinophils (Figure 4A), or mononuclear cells and neutrophils (data not shown).

Using the aforementioned methods, *complex* and *simple* models were constructed for eosinophils, eliciting the equations:
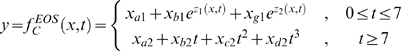
(3)

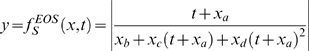
(4)


Although Equations 3 and 4 represent different functions, they both maintain relatively high and similar predictive value, yielding R^2^ values of 0.968 and 0.938, respectively. Using these equations, we performed computer simulations in two- and three-dimensions (Figure 4B–D). These images illustrate that the decrease in airway eosinophilia occurs throughout the entire range of exposures, and that is not dependent on eosinophilia reaching an absolute level; moreover, it is also evident that the higher the eosinophil level, the sooner the downturn begins. This suggests that part of the program of the immune-inflammatory response elicited by chronic allergen exposure may contain an inherent controlling mechanism to prevent persistent eosinophilia in the lung.

### Modeling allergic sensitization to long-term HDM exposure

**Figure 5:**
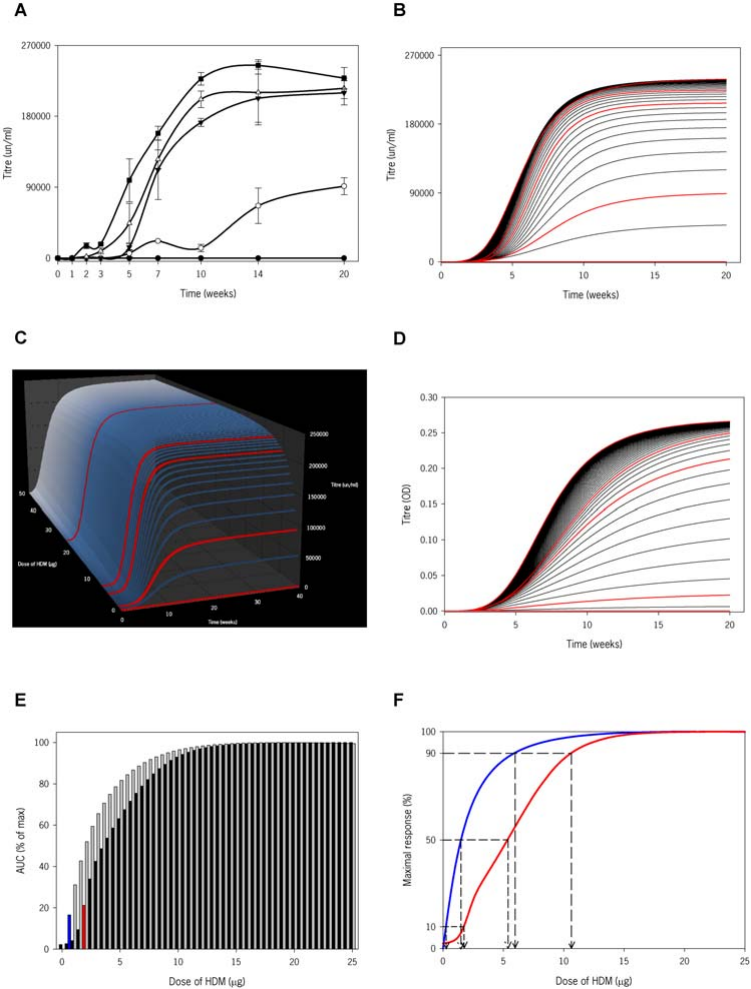
Systemic responses in HDM-exposed mice, subsequent mathematical modeling and comparison between inflammation and sensitization. In panels B, C and D, the simulations for the doses of HDM used experimentally are highlighted in red. (A) Serum levels of HDM-specific IgG_1_. BALB/C mice were exposed to either saline (solid circles) or HDM, 1 µg (open circles), 5 µg (solid triangles down), 7.5 µg (open triangles up) or 25 µg (solid squares) for up to 20 weeks. Data represent mean±s.e.m. (n = 2–9 mice/group). (B) Mathematical model for HDM-specific IgG_1_. A simple mathematical model was developed and IgG_1_ levels over time and at doses ranging from 0 to 25 µg of HDM, in 0.5 µg increments, were predicted. (C) 3D representation of HDM-specific IgG_1_ responses, including predictions up to 50 µg and 40 weeks of HDM exposure. (D) Mathematical model for HDM-specific IgE. A simple mathematical model based on serum measurements was developed and IgE levels were simulated over time and at doses ranging from 0 to 25 µg of HDM in 0.5 µg increments. (E) Area under the curve (AUC) of the maximal number of eosinophils (black bars) and level of HDM-specific IgG_1_ (grey bars). The lower dose showing a change in the behavior of the curve (threshold dose), is identified for HDM-specific IgG_1_ (blue bar, 0.5 µg) and eosinophilia (red bar, 2 µg). The results are based on computer simulations. (F) Maximal responses for HDM-specific IgG_1_ (blue line) and eosinophilia (red line) at a range of doses of HDM. The 90% of the maximal inflammatory or immunoglobulin response (long dashed line) is reached when given about 11 or 6 µg of HDM, respectively; approximately 2 and 5 µg of HDM are required to elicit 50% of the maximal inflammatory and immunoglobulin responses (medium dashed line), and <1 and 2 µg to induce 10% of these responses (short dashed line).

Allergic sensitization is a crucial event in allergic asthma. Hence, we investigated the effect of allergen exposure on defining features of B cell immunity, namely serum levels of HDM-specific immunoglobulins. As shown in Figure 5A–D, IgG_1_ and IgE serum levels follow a logistic-like behavior similar to that identified for total airway inflammation. However, there are kinetic differences; indeed, serum immunoglobulins are detected after 2–3 weeks of allergen exposure at a time where the inflammatory response has already reached its peak. Given the nature of the immunoglobulin response, *simple* models for both IgE and IgG_1_ were constructed and proved to be accurate (R^2^ values of 0.900 and 0.985, respectively). Equations 5 and 6 depict the values of IgE and IgG_1_:
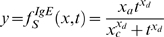
(5)

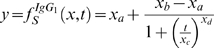
(6)


### Relationship between allergic sensitization and airway inflammation

Airway inflammation and allergic sensitization were compared using two different approaches. First, we considered the *threshold* of responsiveness, understood here as the lowest dose of allergen that elicits a measurable response. To address this, we calculated areas under the curve (AUC) for all modeled responses and determined that a threshold would be the point at which there was an apparent change in behavior. The lowest dose of allergen required to elicit an eosinophilic response is 2 µg, whereas that required to induce an IgG_1_ response is 0.5 µg. (Figure 5E). Of interest, these doses elicit responses that are approximately 20% of the maximal inducible response. It is clear that the pattern of the areas under each curve for IgG_1_ and eosinophils are similar; however, the latter is shifted to the right indicating that the amount of allergen required to elicit not only the lowest response but all responses is different. To better visualize this, and to standardize measurements, we plotted each outcome as a percentage of the maximal response. As shown in Figure 5F, any level of sensitization is achieved with about half the amount of allergen required to achieve the same level of eosinophilic inflammation. Higher IgG_1_ responses are not only induced by the same amount of allergen but also greater changes in IgG_1_ are observed at lower doses of allergen. Similar observations were made for TCN and IgE (data not shown).

### Lung function and remodeling

**Figure 6:**
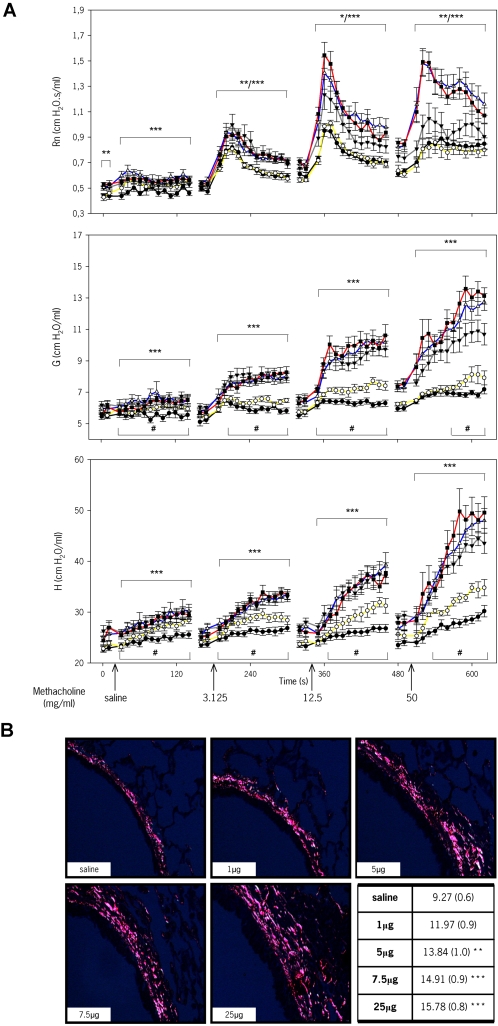
Physiological and structural lung changes in mice exposed to different doses of HDM. (A) Analysis of airway responsiveness to methacholine (MCh) in mice exposed to HDM for 3 weeks. Airway resistance (Rn), tissue dampening (G) and tissue elastance (H) were determined in BALB/C mice exposed to either saline (solid circles, black line) or HDM, 1 µg (open circles, yellow line), 5 µg (solid triangles down, grey line), 7.5 µg (open triangles up, blue line) or 25 µg (solid squares, red line). A time-course of 2 baseline measurements prior to nebulization of increasing doses of MCh (0, 3.125, 12.5 and 50 mg/ml) followed by 12 consecutive measurements is shown. Data represent mean±s.e.m. (n = 5–12 mice/group). *, ** and *** indicate p<0.05, <0.01 and <0.001, respectively, in mice exposed to 5, 7.5 and 25 µg compared to saline; ^#^ indicates p<0.05 in mice exposed to 1 µg compared to saline. (B) Airway remodeling after 7 weeks of HDM exposure. Picro Sirius red-stained lung sections visualized under polarized light (×20) and morphometric analysis show increased subepithelial accumulation of collagen in HDM exposed mice. Data represent the mean of the percentages of stained area of interest (±s.e.m.). ** indicates p<0.01 and ***, p<0.001 versus saline exposed mice (n = 7–12 mice/group).

Airway dysfunction, notably airway hyperreactivity (AHR), is a hallmark of allergic asthma. Preliminarily, we have evaluated lung function to a range of doses after 3 weeks of HDM exposure, a time-point where there is prominent inflammation but no airway remodeling [15]. As shown in Figure 6A, airway resistance (Rn), tissue dampening (G) and elastance (H) increase dose-dependently, being severe in mice exposed to 7.5 and 25 µg of HDM. Most of the peripheral effects observed at these doses of allergen exposure, as measured by G and H, can be explained as airway closure with some elements of lung heterogeneity, as assessed by hysteresivity (η, data not shown) [19]. Rn in mice exposed to 1 µg of HDM was not significantly different than that in saline-treated animals. Interestingly, G and H seemed to be increased in these mice suggesting incipient functional abnormalities occurring prior to detectable inflammation. To note, mice exposed to 5, 7.5 and 25 µg of HDM had a significantly higher baseline Rn compared to the 1 µg and saline groups, indicating a degree of permanent narrowing of the conducting airways. A comprehensive clarification of the variables that influence airway function in this system will require not only the acquisition of an extensive set of functional data but also of additional data including mucous production, permeability and airway structural changes, i.e. remodeling. In specific regard to the later, Figure 6B shows that subepithelial collagen deposition increases in a dose-dependent manner after 7 weeks of allergen exposure; changes in mice exposed to 1 µg of HDM were not significant compared to saline. Clearly, a quantitative delineation of the relationships between tissue and functional variables with inflammatory and immune variables is a major computational challenge beyond the scope of the research presented here.

## Discussion

Understanding immune responsiveness will benefit from accepting the multidimensionality and quantitative nature of immunological phenomena [20]. Here, we have engaged this precept to investigate immune-inflammatory responses following repeated HDM exposure in mice. The computational analysis we have performed allows for the identification of rules and parameters that define the system. Principal rules are that relationships between time and infiltrating total cells, as well as mononuclear cells and neutrophils, and serum immunoglobulins follow a logistic-like curve; in sharp contrast, the eosinophil response over time follows a bell shaped-like curve. These rules presuppose a dynamic behavior with at least one significant implication: the lung cellular effector profile quite drastically changes depending on dose and length of exposure to allergen. These multiple possible outcomes may be mathematically viewed as a demonstration of heterogeneity.

The distinct behavior of eosinophils is intriguing. The underlying immunological explanation is unknown at this time; however, it seems intuitive that if allergen exposure is considered as an input, persistent deliverance of such an input will stress the system and instigate reactive responses. From this perspective, the decrease of eosinophils and the increase in mononuclear cells are likely to be mechanistically related. Flow cytometric analysis delineating the changes in the dynamics of subsets of mononuclear cells (T cells and monocyte/macrophages) over the entire protocol will be informative and suggest future venues of research.

Several parameters define the behavior of the system. First, we have identified a threshold dose at approximately 0.5 µg of HDM for sensitization and 2 µg for inflammation. In fact, exposure to 2.5 µg of HDM elicits a detectable eosinophilic inflammatory response (data not shown). Second, responsiveness for all constituents is dependent on the strength of the initial dose of allergen; moreover, the greater the input, the steeper the initial slope of the response. Third, the system has an inherently limited capacity to respond, at least to the same allergen. This maximal responsiveness is achieved at a dose between 10 and 15 µg, and further increases in dose or length of exposure do not result in greater responses. Fourth, there is an entire range of responses between the threshold and the maximum; mathematically, however, the model reveals that the distribution of responses is non-linear. Lastly, a comparative analysis of inflammation and sensitization outputs reveals that the development of the latter is more sensitive to allergen than the induction of airway inflammation. That the relationships between exposure and either sensitization or inflammation are non-linear intimates that the relationship between sensitization and inflammation is non-linear as well. It is tempting to speculate that these findings may contribute to explain the difference between the prevalence of atopy (∼40%) and asthma (5–10%) in humans [21], [22], [23], [24].

The question of how the concentrations of allergen used here compare to human exposure is elusive because the terms of reference are precarious (reviewed in [25]). Many studies have examined the amount of mite allergen present in homes. However, the numbers vary extraordinarily. Not only is there a plethora of environmental variables influencing the concentration of mite allergens in the household but there are also several collection and measurement techniques [3], [26], [27], [28]. In addition, the relationship between the micrograms of allergen measured in a dust sample and the amount of allergen that is airborne, inspired, and reaches the lower airway is enigmatic. Indeed, the inability to precisely determine mucosal HDM exposure in humans frustrates the justifiable desire to formulate a rigorous interspecies comparison of exposures. Perhaps such a straightforward comparison is an ill-conceived goal; arguably, numbers may not be translated between species but behaviors likely can.

Many issues have not been addressed here. For example, experiments were conducted in BALB/C mice. While we know that C57BL/6 mice respond to HDM even more vigorously in terms of inflammation, it definitely cannot be assumed that the behavior of these two strains, or others, is identical. Similarly, these experiments were performed in female mice and, thus, a direct application to male mice is unadvisable. In addition, we cannot presume that the behaviors described for HDM apply to other aeroallergens. With these limitations, our research furnishes a conceptual foundation and operating tools for the evaluation of other variables or system perturbations of a pharmacological, environmental or genetic nature. Based on the present research, future analysis of immune responses exploring these variables may not require the generation of entire data sets but of selected experiments to generate comparative algorithms to re-define the overall behavior of the system.

There has been a considerable interest by engineers, mathematicians and computer scientists in the application of their skills to modeling biological processes. Over the last few years, biologists have shown an increasing attraction to join in this enterprise. Arguably, the catalyst underlying this initiative has been the recognition that biological processes are, formally, complex processes. As such, efforts to incorporate new conceptual and experimental stratagems must be made to better comprehend them. The development of mathematical modeling based upon research, primarily *in vitro*, examining hemopoiesis and stem cell renewal [29], models of virus-immune dynamics [30] and cancer cell propagation [31] typify these efforts. Particularly in the area of inflammation, agent-based and equation-based models have been established to provide insight into the complex dynamics of this process [32], [33], [34], [35], [36], [37]. However, the research presented in this manuscript is, to our knowledge, the first to investigate the interaction between aeroallergens and the immune system *in vivo* from a computational perspective.

## Supporting Information

Figure S1 Iterations to validate the mathematical model for the inflammatory response. (A) A mathematical equation was developed based on the responses to saline, 1, 7.5 and 25 µg of HDM up to 14 weeks (blue lines). Simulations (red lines) for these doses studied were generated. Then, the equation was used to predict the response to 5 ug, which was subsequently evaluated experimentally (blue line, triangles down). (B) A refined mathematical equation was developed based on the responses to saline, 1, 5, 7.5 and 25 ug of HDM up to 14 weeks (blue lines). Simulations (red lines) for these doses were generated. Then, responses for all doses at 20 weeks were predicted, and these were subsequently evaluated experimentally.
(1.50 MB TIF)

Methods S1 Supplementary methods, including elaboration of mathematical Equations 1 to 6.4 used in the models, validation analysis and area under curves.
(0.11 MB PDF)

## References

[1] Lau S, Illi S, Sommerfeld C, Niggemann B, Bergmann R (2000). Early exposure to house-dust mite and cat allergens and development of childhood asthma: a cohort study. Multicentre Allergy Study Group.. Lancet.

[2] Wahn U, Lau S, Bergmann R, Kulig M, Forster J (1997). Indoor allergen exposure is a risk factor for sensitization during the first three years of life.. J Allergy Clin Immunol.

[3] Platts-Mills TA, Vervloet D, Thomas WR, Aalberse RC, Chapman MD (1997). Indoor allergens and asthma: report of the Third International Workshop.. J Allergy Clin Immunol.

[4] Sporik R, Squillace SP, Ingram JM, Rakes G, Honsinger RW (1999). Mite, cat, and cockroach exposure, allergen sensitisation, and asthma in children: a case-control study of three schools.. Thorax.

[5] Sporik R, Holgate ST, Platts-Mills TA, Cogswell JJ (1990). Exposure to house-dust mite allergen (Der p I) and the development of asthma in childhood. A prospective study.. N Engl J Med.

[6] Martinez FD, Wright AL, Taussig LM, Holberg CJ, Halonen M (1995). Asthma and wheezing in the first six years of life. The Group Health Medical Associates.. N Engl J Med.

[7] Cohen IR (2007). Modeling immune behavior for experimentalists.. Immunol Rev.

[8] Stampfli MR, Wiley RE, Neigh GS, Gajewska BU, Lei XF (1998). GM-CSF transgene expression in the airway allows aerosolized ovalbumin to induce allergic sensitization in mice.. J Clin Invest.

[9] Ohkawara Y, Lei XF, Stampfli MR, Marshall JS, Xing Z (1997). Cytokine and eosinophil responses in the lung, peripheral blood, and bone marrow compartments in a murine model of allergen-induced airways inflammation.. Am J Respir Cell Mol Biol.

[10] Fattouh R, Midence G, Arias K, Johnson JR, Walker TD (2008). TGF-{beta} Regulates House Dust Mite-induced Allergic Airway Inflammation but not Airway Remodeling.. Am J Respir Crit Care Med.

[11] Schuessler TF, Bates JH (1995). A computer-controlled research ventilator for small animals: design and evaluation.. IEEE Trans Biomed Eng.

[12] Hantos Z, Daroczy B, Suki B, Nagy S, Fredberg JJ (1992). Input impedance and peripheral inhomogeneity of dog lungs.. J Appl Physiol.

[13] Leigh R, Ellis R, Wattie J, Southam DS, De Hoogh M (2002). Dysfunction and remodeling of the mouse airway persist after resolution of acute allergen-induced airway inflammation.. Am J Respir Cell Mol Biol.

[14] Cates EC, Fattouh R, Wattie J, Inman MD, Goncharova S (2004). Intranasal exposure of mice to house dust mite elicits allergic airway inflammation via a GM-CSF-mediated mechanism.. J Immunol.

[15] Johnson JR, Wiley RE, Fattouh R, Swirski FK, Gajewska BU (2004). Continuous exposure to house dust mite elicits chronic airway inflammation and structural remodeling.. Am J Respir Crit Care Med.

[16] Anderson AE, Ellis BJ, Weiss JA (2007). Verification, validation and sensitivity studies in computational biomechanics.. Comput Methods Biomech Biomed Engin.

[17] Oberkampf WL, Trucano TG, Hirsch C (2002). Verification, validation, and predictive capability in computational engineering and physics..

[18] Oberkampf WLB, F M (2006). Measures of agreement between computation and experiment: validation metrics.. Journal of Computational Physics.

[19] Lundblad LK, Thompson-Figueroa J, Allen GB, Rinaldi L, Norton RJ (2007). Airway hyperresponsiveness in allergically inflamed mice: the role of airway closure.. Am J Respir Crit Care Med.

[20] Greenspan NS (2007). Conceptualizing immune responsiveness.. Nat Immunol.

[21] Holt PG, Thomas WR (2005). Sensitization to airborne environmental allergens: unresolved issues.. Nat Immunol.

[22] Sporik R, Platts-Mills TA (2001). Allergen exposure and the development of asthma.. Thorax.

[23] Pleis JR, Lethbridge-Cejku M (2006). Summary health statistics for U.S. adults: National Health Interview Survey, 2005.. Vital Health Stat.

[24] Masoli M, Fabian D, Holt S, Beasley R (2004). The global burden of asthma: executive summary of the GINA Dissemination Committee report.. Allergy.

[25] Cates EC, Fattouh R, Johnson JR, Llop-Guevara A, Jordana M (2007). Modeling responses to respiratory house dust mite exposure.. Contrib Microbiol.

[26] Tovey ER, Mitakakis TZ, Sercombe JK, Vanlaar CH, Marks GB (2003). Four methods of sampling for dust mite allergen: differences in ‘dust’.. Allergy.

[27] Custis NJ, Woodfolk JA, Vaughan JW, Platts-Mills TA (2003). Quantitative measurement of airborne allergens from dust mites, dogs, and cats using an ion-charging device.. Clin Exp Allergy.

[28] Sakaguchi M, Inouye S, Sasaki R, Hashimoto M, Kobayashi C (1996). Measurement of airborne mite allergen exposure in individual subjects.. J Allergy Clin Immunol.

[29] Kirkland MA (2004). A phase space model of hemopoiesis and the concept of stem cell renewal.. Exp Hematol.

[30] Perelson AS (2002). Modelling viral and immune system dynamics.. Nat Rev Immunol.

[31] Deisboeck TS, Mansury Y, Guiot C, Degiorgis PG, Delsanto PP (2005). Insights from a novel tumor model: Indications for a quantitative link between tumor growth and invasion.. Med Hypotheses.

[32] Vodovotz Y, Clermont G, Chow C, An G (2004). Mathematical models of the acute inflammatory response.. Curr Opin Crit Care.

[33] Reynolds A, Rubin J, Clermont G, Day J, Vodovotz Y (2006). A reduced mathematical model of the acute inflammatory response: I. Derivation of model and analysis of anti-inflammation.. J Theor Biol.

[34] Vodovotz Y, Chow CC, Bartels J, Lagoa C, Prince JM (2006). In silico models of acute inflammation in animals.. Shock.

[35] Day J, Rubin J, Vodovotz Y, Chow CC, Reynolds A (2006). A reduced mathematical model of the acute inflammatory response II. Capturing scenarios of repeated endotoxin administration.. J Theor Biol.

[36] An G (2001). Agent-based computer simulation and sirs: building a bridge between basic science and clinical trials.. Shock.

[37] Chow CC, Clermont G, Kumar R, Lagoa C, Tawadrous Z (2005). The acute inflammatory response in diverse shock states.. Shock.

