# Supplementary figures and images for: In Vivo-to-In Silico Iterations to Investigate Aeroallergen-Host Interactions
Source: PLoS One. 2008 Jun 11;3(6):e2426. doi: 10.1371/journal.pone.0002426 (PMC2409221; doi:10.1371/journal.pone.0002426)

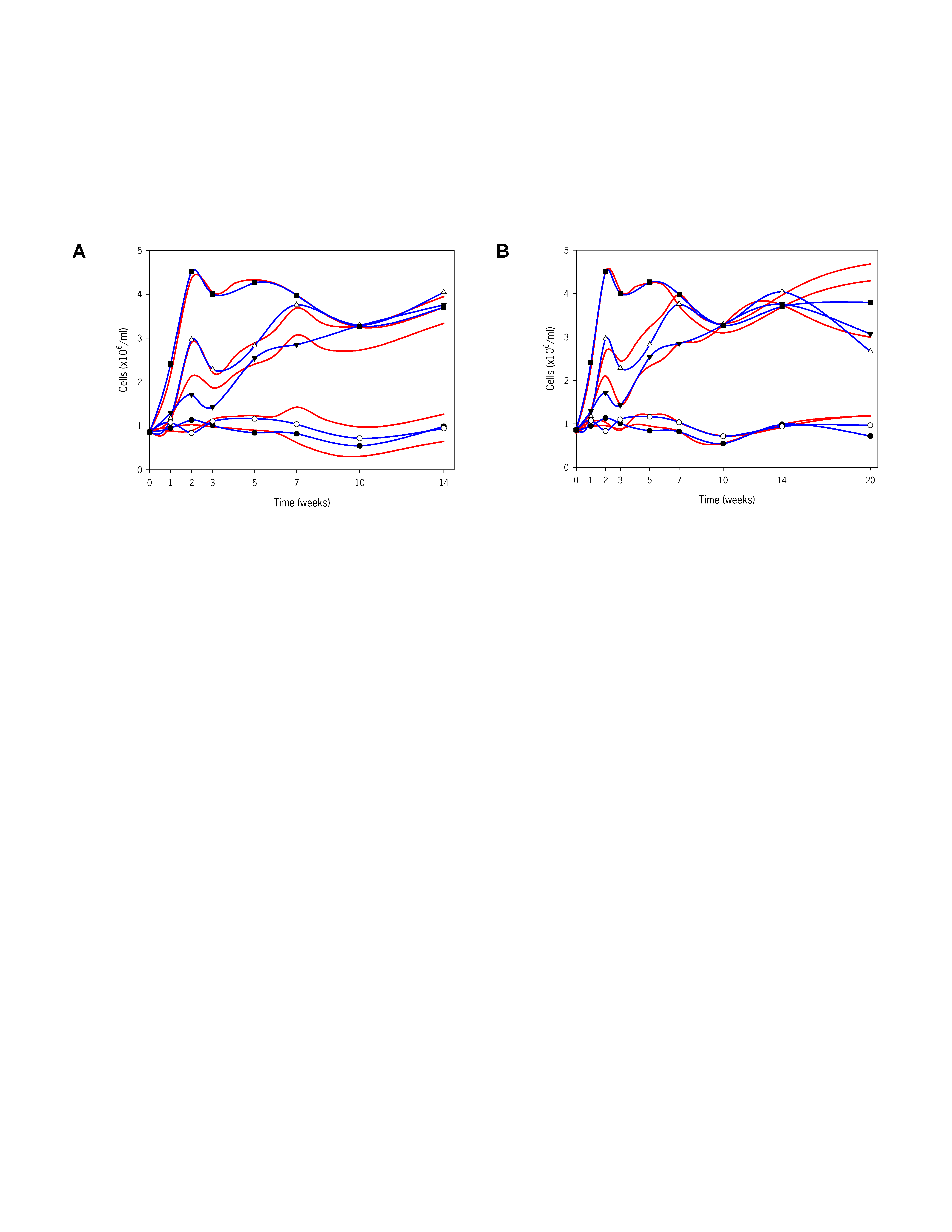

Supplement: Figure S1 — Iterations to validate the mathematical model for the inflammatory response. (A) A mathematical equation was developed based on the responses to saline, 1, 7.5 and 25 µg of HDM up to 14 weeks (blue lines). Simulations (red lines) for these doses studied were generated. Then, the equation was used to predict the response to 5 ug, which was subsequently evaluated experimentally (blue line, triangles down). (B) A refined mathematical equation was developed based on the responses to saline, 1, 5, 7.5 and 25 ug of HDM up to 14 weeks (blue lines). Simulations (red lines) for these doses were generated. Then, responses for all doses at 20 weeks were predicted, and these were subsequently evaluated experimentally. (1.50 MB TIF) [file pone.0002426.s001.tif]
