# Supplementary material for: In Vivo-to-In Silico Iterations to Investigate Aeroallergen-Host Interactions
Source: PLoS One. 2008 Jun 11;3(6):e2426. doi: 10.1371/journal.pone.0002426 (PMC2409221; doi:10.1371/journal.pone.0002426)
Supplement: Methods S1 — Supplementary methods, including elaboration of mathematical Equations 1 to 6.4 used in the models, validation analysis and area under curves. (0.11 MB PDF) [file pone.0002426.s002.pdf]

Methods S1 (Supporting Information)

Mathematical and computational modeling

Outcomes were analyzed by individual doses over specific time periods, deriving a general equation for all doses and all times. Within each equation, numerical coefficients were analyzed based on dose, establishing dose-dependent equations. In some cases, equations were subsequently re-adjusted manually. In order to capture all of the characteristics for a given outcome, *complex* models were derived based on the dynamics of the experimental data. In the case of TCN and EOS, equations were sectioned in parts according to time intervals where it was visually apparent that the curvature of the responses changed; the time-points of 3, 5, and 7 weeks were selected for TCN, and EOS was split at 7 weeks. Through the incorporation of data from additional doses and time-points, the model proved to be modular, i.e. the inclusion of new data did not require the derivation of a new equation, but rather only re-adjustment of the existing model. After performing simulations with the *complex* models, it was determined that *simple* models would yield the general characteristics of each outcome with more clarity and conciseness. Such *simple* models were generated for TCN, EOS, IgG_1 and IgE using a single equation for all time-points, as opposed to defining dose-responses piecewise. 2D comparison of actual data and simulated data was visualized in SigmaPlot software (Systat Software Inc.), while 3D simulation images were generated using Microsoft Excel 2007.

The following sets of equations describe the dynamics of each measured outcome (y) in the context of dose (x, in μg of HDM) and time (t, in weeks).

TCN (Complex)

$$y = f_C^{TCN}(x,t) = \begin{cases} x_{a1} + x_{b1} sin(z_1(x,t))^2 & , \quad 0 \le t \le 3 \\ x_{a2} + x_{b2} e^{z_2(x,t)} + x_{g2} t + x_{h2} t^2 & , \quad 3 \le t \le 7 \\ x_{a3} + x_{b3} e^{z_3(x,t)} + x_{g3} e^{z_4(x,t)} & , \quad t \ge 7 \end{cases} \qquad (1)$$

where,

$$z_1 = \frac{\pi(t - x_{c1})}{x_{d1}} \tag{1.1}$$

$$z_2 = -0.5\left(\frac{t - x_{c2}}{x_{d2}}\right)^2 \tag{1.2}$$

$$z_3 = -0.5\left(\frac{t - x_{c3}}{x_{d3}}\right)^2 \tag{1.3}$$

$$z_4 = -\left(\frac{t - x_{h2}}{x_{k2}}\right) \tag{1.4}$$

$$x_{a1} = 1.172 - 0.516e^{-0.5\left(\frac{x-12.955}{12.284}\right)^2} \tag{1.5}$$

$$x_{b1} = -0.245 + \frac{4.171}{\left(1 + 10^{-0.103(x+0.252)}\right)^{3.802}} \tag{1.6}$$

$$x_{c1} = 0.029 + 0.377\sin\left(\pi\left(\frac{x - 0.066}{24.889}\right)\right)^2 \tag{1.7}$$

$$x_{d1} = \frac{3.361 + 0.289x + 0.004x^2 - 0.178x^3}{1 + 0.280x + 0.0002x^2 - 0.037x^3} \tag{1.8}$$

$$x_{a2} = \begin{cases} 0.996 - 0.056x + 0.049\left(0.377^x\right) & , \quad 0 \le x \le 5 \\ 5.338\left(1 - e^{-\left(0.065(x-4.382)\right)^{0.606}}\right) & , \quad x > 5 \end{cases} \tag{1.9}$$

$$x_{b2} = \begin{cases} -0.055 + 0.229x - 0.007x^2 & , \quad 0 \le x \le 5 \\ -0.039 + \dfrac{0.958}{1 + \left(\frac{x-5.050}{10.092}\right)^{0.167}} & , \quad x > 5 \end{cases} \tag{1.10}$$

$$x_{c2} = \begin{cases} \dfrac{x+0.157}{0.006+0.220(x+0.157)-0.005(x+0.157)^2} & , \quad 0 \le x \le 5 \\ 5.208-\dfrac{9.888}{x}+\dfrac{47.078}{x^2} & , \quad x > 5 \end{cases} \tag{1.11}$$

$$x_{d2} = \begin{cases} 1.166+\dfrac{0.948}{1+\left(\frac{x}{2.152}\right)^{0.438}} & , \quad 0 \le x \le 5 \\ \dfrac{x-4.562}{0.103+0.401(x-4.562)+0.013(x-4.562)^2} & , \quad x > 5 \end{cases} \tag{1.12}$$

$$x_{g2} = \begin{cases} 0.014+0.002x+0.0001x^2 & , \quad 0 \le x \le 5 \\ 0.149-\dfrac{3.067}{x}+\dfrac{12.508}{x^2} & , \quad x > 5 \end{cases} \tag{1.13}$$

$$x_{h2} = \begin{cases} -0.007+0.004x+0.0006x^2 & , \quad 0 \le x \le 5 \\ 0.039+0.002x^{0.524}-0.0004x^{1.478} & , \quad x > 5 \end{cases} \tag{1.14}$$

$$x_{a3} = \begin{cases} 0.298+0.657x-0.020x^2 & , \quad 0 \le x \le 5 \\ \dfrac{x-4.749}{-0.024+0.423(x-4.749)-0.008(x-4.749)^2} & , \quad x > 5 \end{cases} \tag{1.15}$$

$$x_{b3} = \begin{cases} -0.455-0.216\sqrt{x}+0.347x & , \quad 0 \le x \le 5 \\ -5.056+6.237e^{\frac{-(\ln(x)-2.075)^2}{3.487}} & , \quad x > 5 \end{cases} \tag{1.16}$$

$$x_{c3} = \begin{cases} \dfrac{9.212+5.586x}{1+0.431x-0.013x^2} & , \quad 0 \le x \le 5 \\ 3.101+10.475e^{\frac{-(\ln(x)-2.033)^2}{3.914}} & , \quad x > 5 \end{cases} \tag{1.17}$$

$$x_{d3} = \begin{cases} 4.064+1.785e^{\frac{-x}{1.797}}-2.582e^{\frac{-x}{5.497}}-1.547e^{\frac{-x}{6.004}} & , \quad 0 \le x \le 5 \\ \dfrac{x-4.505}{-0.194+0.808(x-4.505)-0.016(x-4.505)^2} & , \quad x > 5 \end{cases} \tag{1.18}$$

$$x_{g3} = \begin{cases} 0.019 - 0.066x + 0.739\left(0.246^{x}\right) & , \quad 0 \le x \le 5 \\ -0.316 + 6.708e^{\frac{-(x-5.078)}{9.779}}\left(1 - e^{\left(\frac{-(x-5.078)}{13.719}\right)^{0.489}}\right) & , \quad x > 5 \end{cases} \tag{1.19}$$

$$x_{h3} = \begin{cases} 4.527 - 1.986e^{\frac{-x}{0.213}} + 3.123e^{\frac{-x}{9.057}} & , \quad 0 \le x \le 5 \\ 1.127\left(\dfrac{x}{-3.863 + x}\right) + 3.257\left(\dfrac{x}{28.574 + x}\right) + 0.178x & , \quad x > 5 \end{cases} \tag{1.20}$$

$$x_{k3} = \begin{cases} 5.291 + 30.559e^{-3.157x} & , \quad 0 \le x \le 5 \\ 7.844 + \dfrac{-2.847}{1 + \left(\frac{x-4.997}{9.340}\right)^{0.269}} & , \quad x > 5 \end{cases} \tag{1.21}$$

TCN (Simple)

$$y = f_S^{TCN}(x,t) = \frac{x_a + x_b t + x_c t^2}{1 + x_d t + x_g t^2} \tag{2}$$

where,

$$x_a = 0.989 + 0.046\left(\frac{0.665}{1 + 10^{0.407(x-6.214)}}\right) + 0.045\left(\frac{1 - 0.665}{1 + 10^{-0.097(x-18.773)}}\right) - 1.4 \tag{2.1}$$

$$x_b = 0.187 + 1.091\left(\frac{0.978}{1 + 10^{-0.571(x-6.389)}}\right) + 1.091\left(\frac{1 - 0.978}{1 + 10^{-0.081(x-18.780)}}\right) \tag{2.2}$$

$$x_c = 0.378 + 1.137e^{-0.5\left(\frac{x-7.548}{0.595}\right)^2} - 0.239e^{-\left(\frac{x-0.876}{12.035}\right)} \tag{2.3}$$

$$x_d = 0.009 + 1.995\left(\frac{1}{1 + e^{-\frac{x-0.638}{0.273}}}\right)\left(1 - \frac{1}{1 + e^{-\frac{x-0.638}{41.471}}}\right) \tag{2.4}$$

$$x_g = 0.142 + 0.473e^{-0.5\left(\frac{x-7.155}{1.063}\right)^2} + 0.050e^{-\left(\frac{x-0.326}{12.839}\right)} \tag{2.5}$$

EOS (Complex)

$$y = f_C^{EOS}(x,t) = \begin{cases} x_{a1} + x_{b1}e^{z_1(x,t)} + x_{g1}e^{z_2(x,t)} & , \quad 0 \le t \le 7 \\ x_{a2} + x_{b2}t + x_{c2}t^2 + x_{d2}t^3 & , \quad t \ge 7 \end{cases} \tag{3}$$

where,

$$z_1 = -0.5\left(\frac{t - x_{c1}}{x_{d1}}\right)^2 \tag{3.1}$$

$$z_2 = -0.5\left(\frac{t - x_{h1}}{x_{k1}}\right)^2 \tag{3.2}$$

$$x_{a1} = \frac{-0.018 + 0.106x - 0.145x^2 - 0.005x^3}{1 + 0.096x - 0.151x^2 - 0.005x^3} \tag{3.3}$$

$$x_{b1} = \frac{0.057x^{1.633}}{1 + 0.036x^{1.633}} \tag{3.4}$$

$$x_{c1} = 2.595 - 2.021e^{\frac{-x}{10.062}} + 2.935e^{\frac{-x}{32.085}} \tag{3.5}$$

$$x_{d1} = \frac{7.088 + 0.903x - 0.199x^2}{1 + 0.048x - 0.113x^2} \tag{3.6}$$

$$x_{g1} = \frac{0.045x^{2.665}}{1 + 0.031x^{2.665}} \tag{3.7}$$

$$x_{h1} = 10.578 + 0.234x - 0.019x^2 + 0.0004x^3 \tag{3.8}$$

$$x_{k1} = 7.078 - 0.097\sqrt{x} + 0.076x \tag{3.9}$$

$$x_{a2} = -0.233\left(\frac{x}{0.553 + x}\right) + 0.508\left(\frac{x}{4.993 + x}\right) + 0.176x \tag{3.10}$$

$$x_{b2} = \begin{cases} -0.066 + \dfrac{0.110}{1 + 10^{0.232x - 0.109}} & , \quad 0 \le x \le 5 \\ 0.335 - 0.019x & , \quad x > 5 \end{cases} \tag{3.11}$$

$$x_{c2} = \frac{x}{512.820 + 205.158x + 324.354\sqrt{x}} \tag{3.12}$$

$$x_{d2} = 2.590E^{-06} - 1.509E^{-05}x + 5.980E^{-07}x^2 \tag{3.13}$$

EOS (Simple)

$$y = f_S^{EOS}(x,t) = \left| \frac{t + x_a}{x_b + x_c(t + x_a) + x_d(t + x_a)^2} \right| \tag{4}$$

where,

$$x_a = -0.047 - 0.019\ln(x + 0.081) \tag{4.1}$$

$$x_b = 3.737 + 46.203e^{\frac{-(x-0.180)}{2.148}} \left| 1 - e^{\frac{-(x-0.180)}{12.861}} \right|^{0.145} \tag{4.2}$$

$$x_c = -0.615 + 5.029e^{-0.987x} \tag{4.3}$$

$$x_d = 0.200 + \frac{7.909}{1 + 10^{1.439x - 1.389}} \tag{4.4}$$

IgE (Simple)

$$y = f_S^{IgE}(x,t) = \frac{x_a t^{x_d}}{x_c^{\,x_d} + t^{x_d}} \tag{5}$$

where,

$$x_a = 0.272\left(1 - e^{(-0.297(x-0.015))^{1.819}}\right) \tag{5.1}$$

$$x_c = 6.737 + 70.018\left(\frac{20.601}{4(x-1.682)^2 + 20.601^2}\right) \tag{5.2}$$

$$x_d = \frac{17.942 + 9.965x^{1.562}}{9.111 + x^{1.562}} \tag{5.3}$$

IgG₁ (Simple)

$$y = f_S^{IgG_1}(x,t) = x_a + \frac{x_b - x_a}{1 + \left(\frac{t}{x_c}\right)^{x_d}} \tag{6}$$

where,

$$x_a = \frac{x}{1.06E^{-05} + 4.583E^{-06}x - 4.237E^{-06}\sqrt{x}} \tag{6.1}$$

$$x_b = \frac{-244.738x}{1.527 + x} \tag{6.2}$$

$$x_c = 5.752 + \frac{4.489}{1 + 10^{0.855(x-1.261)^{0.182}}} \tag{6.3}$$

$$x_d = 2.133 + \frac{7.243x}{1.915 + x} + \frac{-8.133x}{16.102 + x} \tag{6.4}$$

---

*Note: All numerical coefficients above are given to 3 decimal places for brevity, however all equations were computed using the full coefficients as derived by the model.

Validation analysis

Linear regression analysis and 95% CI were used to validate the mathematical models since they are deterministic and non-deterministic approaches, respectively [1]. *Deterministic validation metric:* Actual data was plotted against model data and linear regression performed with the condition that the regression line must pass through (0,0). The possible goodness of fit statistic, R^2 (coefficient of determination), was calculated. An R^2 of 1 would mean that all values of actual data and model data are equal and thus lie upon the line y=x. *Non-deterministic validation metric:* 95% CIs were created using the appropriate *t* probability distribution ($t_{0.05,\ df}$), depending on the number of degrees of freedom (number of mice sacrificed minus 1) at each time (t) and dose (x) with an α of 0.05. In addition, as proposed by Oberkampf, *et al.* [2], global validation metrics to quantify overall model accuracy were calculated as follows:

$$\text{Average Relative Accuracy of Model } = 1 - \left[\frac{1}{x_{\max}} \int_0^{25} \frac{1}{t_{final}} \int_0^{t_{final}} \left| \frac{\overline{y_a} - y_m}{\overline{y_a}} \right| dtdx \right]$$

where y_a is the actual mean (obtained from experiment), y_m is the predicted mean (virtual data). Definite integrals, estimated by trapezoidal Riemann sum, are used instead of straight summation in order to be able to assess predictive (interpolation) capabilities of each model. A similar method is applied to calculate the confidence indicator (half-width 95% CIs are averaged over both time and dose):

$$\text{Confidence Indicator} = \left[\frac{1}{x_{\max}} \int_0^{x_{\max}} \frac{1}{t_{final}} \int_0^{t_{final}} \frac{SD}{\sqrt{n_{t,x}}} \frac{t_{0.05,df}}{\overline{y_a}} dtdx \right]$$

The average relative accuracy of the model, in line with assessment using linear regression, respectively calculated our initial, second and final *complex* TCN models to be 94.23±21.55%, 95.49±22.67% and 97.50±22.42% accurate (to a maximum of a 100%) with 95% confidence. Comparing between *complex* and *simple* TCN models, the *complex* TCN model again provides slightly more accurate predictions than the *simple* model, 97.50±22.42% versus 89.03±22.42% accuracy with 95% confidence. Further, our models (both *simple* and *complex*) fall within the 95% CI band meaning that there is a high

probability for our model to be able to predict real responses. Comprehensive non-deterministic analysis supports our findings from linear regression and allows for inferences to be made about our models' predictive capabilities.

Area under curves

For each outcome (IgG_1 and EOS), equations that represented each increment of 0.5μg HDM, dose-response curves were exported into Wolfram Mathematica software (Wolfram Research Inc.) to calculate the definite integral from 0 to 20 weeks of each equation.

References

1. Anderson AE, Ellis BJ, Weiss JA (2007) Verification, validation and sensitivity studies in computational biomechanics. Comput Methods Biomech Biomed Engin 10: 171-184.

2. Oberkampf WLB, M.F. (2006) Measures of agreement between computation and experiment: validation metrics. Journal of Computational Physics 217: 5-36.
